# Non-invasive imaging of sympathetic innervation of the pancreas in individuals with type 2 diabetes

**DOI:** 10.1007/s00125-023-06039-7

**Published:** 2023-11-07

**Authors:** Achyut Ram Vyakaranam, Maryama M. Mahamed, Per Hellman, Olof Eriksson, Daniel Espes, Gustaf Christoffersson, Anders Sundin

**Affiliations:** 1 Department of Surgical Sciences, Section of Radiology & Molecular Imaging, Uppsala University, Uppsala, Sweden; 2 Science for Life Laboratory, Department of Medicinal Chemistry, Uppsala University, Uppsala, Sweden; 3 Science for Life Laboratory, Department of Medical Cell Biology, Uppsala University, Uppsala, Sweden; 4 Science for Life Laboratory, Department of Medical Sciences, Uppsala University, Uppsala, Sweden

**Keywords:** 11C-HED, 11C-hydroxy ephedrine, A41, Diabetes, Innervation, PET-CT specific binding index

## Abstract

**Aims/hypothesis:**

Compromised pancreatic sympathetic innervation has been suggested as a factor involved in both immune-mediated beta cell destruction and endocrine dysregulation of pancreatic islets. To further explore these intriguing findings, new techniques for in vivo assessment of pancreatic innervation are required. This is a retrospective study that aimed to investigate whether the noradrenaline (norepinephrine) analogue ^11^C-hydroxy ephedrine (^11^C-HED) could be used for quantitative positron emission tomography (PET) imaging of the sympathetic innervation of the human pancreas.

**Methods:**

In 25 individuals with type 2 diabetes and 64 individuals without diabetes, all of whom had previously undergone ^11^C-HED-PET/CT because of pheochromocytoma or paraganglioma (or suspicion thereof), the ^11^C-HED standardised uptake value (SUV_mean_), ^11^C-HED specific binding index (SBI), pancreatic functional volume (FV, in ml), functional neuronal volume (FNV, calculated as SUV_mean_ × FV), specific binding index with functional volume (SBI FV, calculated as SBI × FV) and attenuation on CT (HU) were investigated in the entire pancreas, and additionally in six separate anatomical pancreatic regions.

**Results:**

Generally, ^11^C-HED uptake in the pancreas was high, with marked individual variation, suggesting variability in sympathetic innervation. Moreover, pancreatic CT attenuation (HU) (*p*<0.001), ^11^C-HED SBI (*p*=0.0049) and SBI FV (*p*=0.0142) were lower in individuals with type 2 diabetes than in individuals without diabetes, whereas ^11^C-HED SUV_mean_ (*p*=0.15), FV (*p*=0.73) and FNV (*p*=0.30) were similar.

**Conclusions/interpretation:**

We demonstrate the feasibility of using ^11^C-HED-PET for non-invasive assessment of pancreatic sympathetic innervation in humans. These findings warrant further prospective evaluation, especially in individuals with theoretical defects in pancreatic sympathetic innervation, such as those with type 1 diabetes.

**Graphical Abstract:**

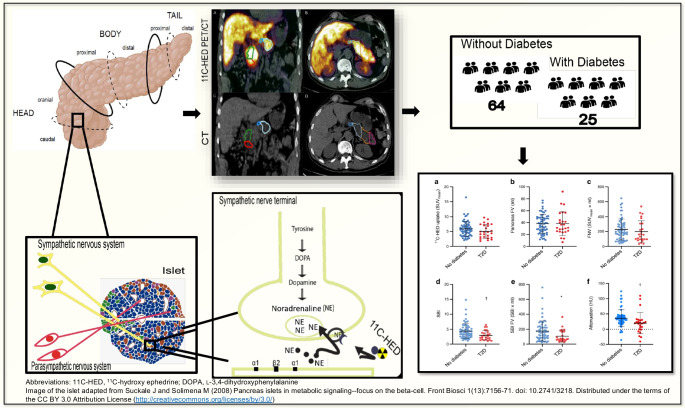

**Supplementary Information:**

The online version of this article (10.1007/s00125-023-06039-7) contains peer-reviewed but unedited supplementary material.


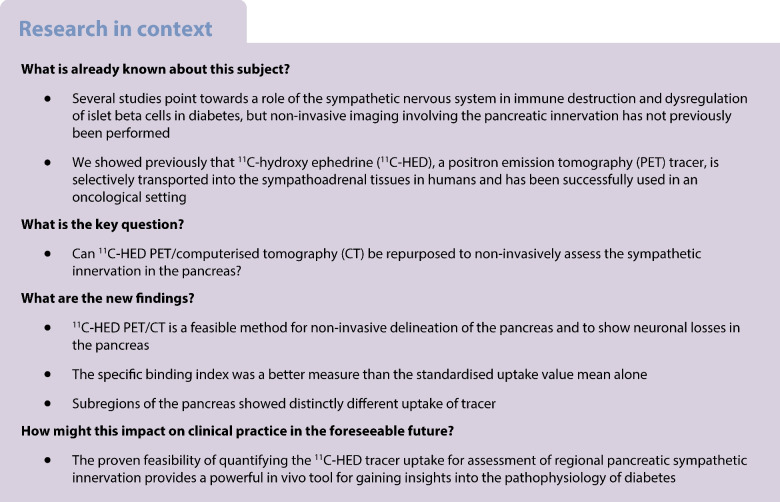


## Introduction

Diabetes constitutes a large and growing health concern worldwide, and the exact aetiologies of human type 1 diabetes and type 2 diabetes still remain unclear. Understanding the early events that lead to pancreatic beta cell dysfunction and destruction might provide insights and options for novel therapeutic interventions in these diseases [1, 2]. In both type 1 diabetes and type 2 diabetes, nerve signalling to the pancreas has been proposed to influence immune events and endocrine homeostasis [3, 4]. Several studies report that the pancreas is richly innervated by the autonomous nervous system and that sensory neurons are also present within the islets [5–9]. Interestingly, disease-related changes in sympathetic innervation mostly seem to be associated with the pancreatic islets [3, 10, 11], while changes in parasympathetic nerves mainly seem to be associated with the exocrine pancreas [12].

Although pancreatic islets can respond to changes in blood glucose levels autonomously (e.g. isolated in culture), increasing evidence suggests that neural control of the endocrine pancreas is important for fine tuning this response when homeostasis is challenged, such as during pregnancy and obesity [13]. Sympathetic signalling to the pancreatic islets promotes the secretion of glucagon from the alpha cells and inhibits the release of insulin from the beta cells [14]. Failure to adapt to changes in insulin sensitivity may lead to beta cell dysfunction, which is why the autonomic nervous system has been hypothesised to play an important role in the intricate mechanisms that lead to beta cell dysfunction and, in extension, type 2 diabetes [15].

Dysregulation of islet sympathetic innervation in human type 1 diabetes has been observed in donated pancreases [10]. Moreover, a recent study found decreased sympathetic islet innervation in autoantibody positive individuals, indicating a role for these neurons in the onset of disease [16]. There is also experimental evidence supporting the role of sympathetic islet innervation in the autoimmune process of type 1 diabetes. Specifically, sympathetic innervation seems to be involved in local regulation of immune cell activation and may be responsible for the autoimmune component of rodent models of type 1 diabetes [3, 10, 11, 17].

The need for further evidence of pancreatic sympathetic innervation, and its association with beta cell function and possibly their destruction in humans, necessitates the development and validation of non-invasive in vivo techniques that allow repeated and safe quantification of markers of innervation [18].

Positron emission tomography (PET) is a biomedical imaging modality, well established in the clinical oncological, neurological and cardiovascular settings. PET allows for quantitative, minimally invasive imaging of receptors and enzymatic processes, given the access to suitable radiotracers. The PET radiopharmaceutical ^11^C-hydroxy ephedrine (^11^C-HED) is a noradrenaline (norepinephrine) analogue, which has been extensively used in recent years for clinical assessment of sympathetic innervation of, for example, the myocardium [19–21].

Importantly, noradrenaline transporter receptor (NET)-mediated ^11^C-HED retention in the pancreas has also been demonstrated in animal models, indicating that the pancreatic sympathetic neurons can be imaged by PET [22]. Our experience with the tracer encouraged us to calculate the specific binding index (SBI), which is a widely used concept in quantification, with left ventricular blood as a reference tissue [23–26]. In a previous study on ^11^C-HED-PET/computerised tomography (CT) imaging of adrenal tumours, we further observed a strong accumulation in the pancreas [22, 23]. However, this interesting finding has not been further evaluated in the clinical setting.

The possible involvement of the autonomic sympathetic nervous system in both type 1 diabetes and type 2 diabetes prompted us to evaluate ^11^C-HED-PET/CT as a technique for studying human pancreatic innervation. By quantifying the total and the regional pancreatic uptake of ^11^C-HED in individuals who had previously undergone PET/CT because of pheochromocytoma (PCC) and paraganglioma (PGL), or suspicion thereof, we assessed the feasibility of this tracer for pancreatic imaging. Because a subgroup of these individuals (25/89, 28%) also had a type 2 diabetes diagnosis, this provided an opportunity for comparisons with people without diabetes in the cohort.

## Methods

### Participants

This retrospective single centre study included individuals who underwent ^11^C-HED-PET/CT between January 2005 and December 2020 at the PET centre, Uppsala University Hospital, Uppsala, Sweden. Individuals were referred to the PET centre from Uppsala University Hospital and Karolinska University Hospital, Stockholm, Sweden for the investigation of PCC, PGL or suspicion thereof. Clinical and imaging follow-up data were obtained from the hospital’s picture archive and retrieval system (PACS), radiological information system (RIS) and digital patient record system.

We identified 100 individuals examined by ^11^C-HED-PET in total, but the examinations for 11 individuals had to be excluded due to missing clinical data (*n*=1), children (*n*=2), pancreatic tumour (*n*=2) or inferior image quality not allowing for delineation of the pancreas (*n*=6). The remaining 89 individuals were further analysed based on type 2 diabetes diagnosis (individuals without diabetes [*n*=64] and individuals with an ICD-10 [http://apps.who.int/classifications/icd10/browse/2016/en] type 2 diabetes diagnosis [*n*=25]), and lack of type 1 diabetes diagnosis. Eight individuals with type 2 diabetes were on non-pharmacological treatment (i.e. lifestyle and diet changes), eight individuals received oral glucose-lowering drugs, and nine were treated with exogenous insulin.

The study participants closely mirror the broader population of Sweden in terms of demographics, maintaining a 1:1 male-to-female ratio. The participants are predominantly of European descent, aligning with the country's ethnic composition. Their median age of 59 years closely reflects the typical age distribution within Sweden. Additionally, these individuals reside in Sweden and receive healthcare treatment under the Swedish healthcare system.

### ^11^C-HED-PET examination

Individuals were examined on a GE Discovery ST PET/CT scanner (General Electric Medical Systems, Milwaukee, WI, USA) and from 2016 onwards on digital time-of-flight General Electric Discovery MI digital PET/CT scanner (GE Healthcare), producing 47 slices with a 157 mm axial and a 700 mm trans-axial field of view. The individuals were injected intravenously with a mean dose of 465 MBq (range 193–1093 MBq) ^11^C-HED. Twenty minutes after the tracer injection, a static whole-body examination was acquired from the base of the skull to the upper thighs. A non-contrast-enhanced, low-radiation-dose CT examination was performed for attenuation correction and anatomical correlation.

All PET images were reconstructed into a 128×128 matrix, using ordered subset expectation maximisation (OSEM; two iterations, 21 subsets), applying a 4.29 mm post-processing filter. The PET data were normalised and corrected for dead time, random coincidences, physical decay, scatter and attenuation based on the low-dose CT. The spatial resolution was approximately 6.5 mm. Further details of the PET/CT examinations are given in the electronic supplementary material (ESM methods) data provided. All images were recalculated to show the standardised uptake value (SUV) by dividing the radioactivity concentration in each pixel (Bq/ml) by the injected amount of ^11^C-HED (Bq) per body weight (g), thereby standardised for activity and distribution volume (body weight). For detailed methods, please refer to the ESM Methods.

### Delineation of pancreatic regions

**Fig. 1:**
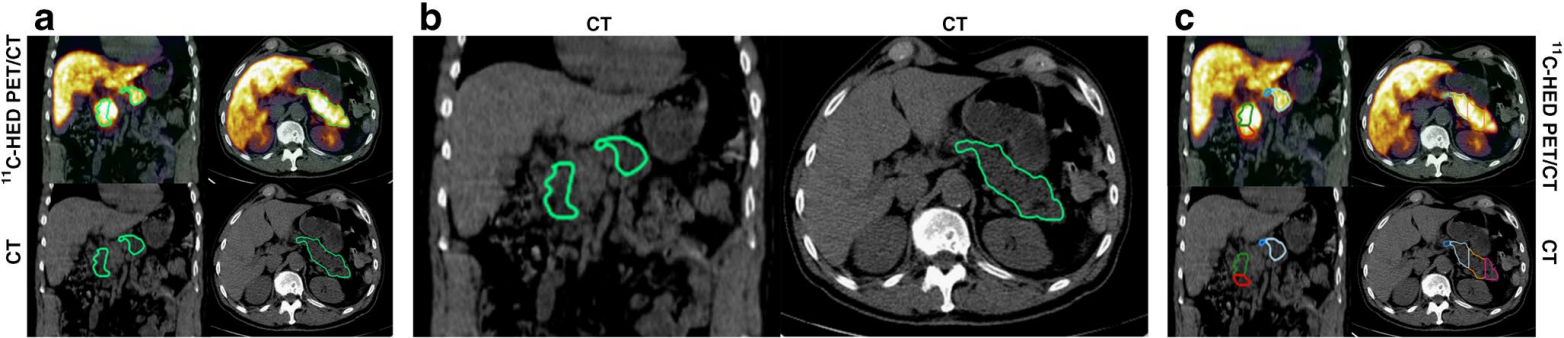
(**a**) ^11^C-HED-PET/CT and CT scans showing delineation of the whole pancreas, applying 41% of the maximum activity cut-off VOI (outlined in green). (**b**) Division of the pancreas was performed manually in the coronal and transversal planes on CT scans to obtain six pancreatic regions. (**c**) Uptake parameters were obtained from the pancreatic regions on ^11^C-HED-PET/CT and CT. Red outlining indicates head caudal; green outlining indicates head cranial; blue outlining indicates body proximal; white outlining indicates body distal; orange outlining indicates tail proximal; and pink outlining indicates tail distal

The pancreas was delineated in the ^11^C-HED-PET/CT examinations, using the Affinity Viewer software v1.0 (Hermes Medical Solutions, Stockholm, Sweden). This software allows the user to display the PET images and corresponding CT images in various anatomical planes, regularly in the transversal, coronal and sagittal planes. The whole pancreas was delineated semi-automatically in the transversal images, using one of the software tools to obtain a 3D volume of interest (VOI). A 3D iso-contour at 41% of the maximum pixel value, adjusted for the background, was obtained as a VOI (Fig. 1a) [27]. A 41% cut-off is routinely used in [^18^F]fluorodeoxyglucose (FDG)-PET analyses and was tested here as well. This cut-off correlated well to the size of the pancreas on CT and was therefore used in the current study. The VOI provided both the mean uptake in the pancreas (SUV_mean_) and the functional volume (FV), which were multiplied (SUV_mean_ × FV) to produce the functional neuronal volume (FNV). The FNV is assumed to indicate the total PET tracer content in a tissue, i.e. the total sympathetic innervation in the pancreas. The 41% iso-contour VOI was also transferred to the CT image volume to achieve the mean attenuation (measured in HU) of the pancreas.

Also, as normal tissue references, a circular region of interest (ROI) of 5 mm diameter was drawn in the left ventricle blood volume from fused PET/CT images, and the mean value of the ROI (SUV_mean_) was registered. We chose the larger blood volume of the left ventricle over the aorta to minimise potential errors in the quantification. Moreover, the ROIs were delineated from fused PET/CT images, and the size was kept small in order to place this well within the blood volume fused PET/CT images to avoid, as far as possible, measurement errors, such as spillover of the signal from the ventricular wall.

The SUV_mean_ of the pancreas was divided by the SUV_mean_ of the blood in the left ventricle to obtain the SBI. Thereby, we were able to calculate the product of SBI and FV as SBI FV.

The pancreas was divided into six anatomical regions defined on the CT: the head (caudal and cranial), body (proximal and distal) and the tail (proximal and distal), which were delineated manually.

In the coronal plane, the head was divided into two parts: caudal and cranial at the level of the superior mesenteric artery (SMA) arising from the aorta (Fig. 1b). The remaining regions were all defined in the transversal plane. The pancreatic body was split from the pancreatic head at the neck, corresponding to a sagittal plane passing through the superior mesenteric vein (SMV), and the pancreatic body proximal extended from the SMV to the lateral wall of the aorta. The pancreatic distal body was delineated from the lateral wall of the aorta to approximately 3 cm to the left. The pancreatic proximal tail extended from 3 to 6 cm from the lateral wall of the aorta. Finally, the last region was the pancreatic distal tail, which reached from 6 cm of the lateral wall of the aorta to the tip of the pancreatic tail (Fig. 1c).

### Statistical methods

Statistical comparisons utilised histograms, the Mann–Whitney U test and a mixed effects model for repeated data applied on ranked data (R, version 4.1.0, R Foundation for Statistical Computing, Austria). Observations recorded as zero were removed from the analyses performed, and the resulting analyses were referred to as ‘with complete observations’ in the text. Moreover, in these analyses, observations were transformed to ranked data to account for severe outliers in the extracted data. All other analyses performed included all available observations. A *p* value of less than 0.05 was considered statistically significant. All values are presented as mean ± SEM.

We were not able to delineate the distal pancreatic tail in more than half of the individuals (56%) because of the pancreatic anatomy. To solve this problem, we subdivided the participants into two groups: individuals with a pancreas comprising all six regions (without diabetes *n*=36, type 2 diabetes *n*=14) and individuals with five regions (without diabetes *n*=60, type 2 diabetes *n*=24). This, however, led to less statistical power in the analysis, based on all six pancreatic regions. Further, because of the missing data for region six, we had to apply a mixed effects model, instead of using the more established ANOVA.

First, the differences in ^11^C-HED uptake (SUV_mean_, SBI), CT attenuation (HU) and the FV (in ml) for the whole pancreas and for all the pancreatic regions were compared between the individuals without diabetes and individuals with type 2 diabetes using a Mann–Whitney *U* test (ESM Tables 1 and 2). Differences between the groups and subgroups were also analysed by the Mann–Whitney *U* test. Furthermore, ^11^C-HED uptake (SUV) and CT attenuation (HU) were compared in individuals with type 2 diabetes, subdivided into two groups: those with a ‘small pancreatic volume’ (SPV), defined as less than the median volume (≤31.4 ml, *n*=13) and those with a ‘large pancreatic volume’ (LPV) (>31.4 ml, *n*=12), respectively (ESM Table 3). In addition, Spearman correlations were computed to measure the strength of association between CT attenuation (HU), FV and the main imaging outcomes, SUV_mean_, SBI, FNV and SBI FV (ESM Tables 4 and 5).

### Ethics considerations

This study was approved by the regional ethics committee (2015/180). All procedures were performed in accordance with the 1964 Declaration of Helsinki and its later amendments or comparable ethical standards. This study constituted a retrospective evaluation of previously collected image data, and without any patient intervention. All study participants gave informed consent.

## Results

Baseline clinical characteristics of all individuals are summarised in Table 1.

**Table 1:** Baseline clinical characteristics of the studied populations

Baseline clinical characteristics	Individuals without diabetes	Individuals with T2D
Total no. of individuals	64	25
Median age, years (range)	57 (18–79)	64 (45–80)
Male-to-female ratio	37:27	9:16
With PCC or PGL diagnosis, *n* (%)	28 (44)	13 (52)
Without PCC or PGL diagnosis, *n* (%)	36 (56)	12 (48)
Years since T2D diagnosis, mean (range)		
Whole T2D cohort (*n*=25)	–	7.7 (0–29.5)
Individuals treated with exogenous insulin or glucose-lowering drugs (*n*=17)	–	6.9 (0–20)
Individuals prescribed changes in lifestyle and diet (*n*=8)	–	9.2 (0.4–29.5)

T2D, type 2 diabetes

### ^11^C-HED uptake in the pancreas

**Fig. 2:**
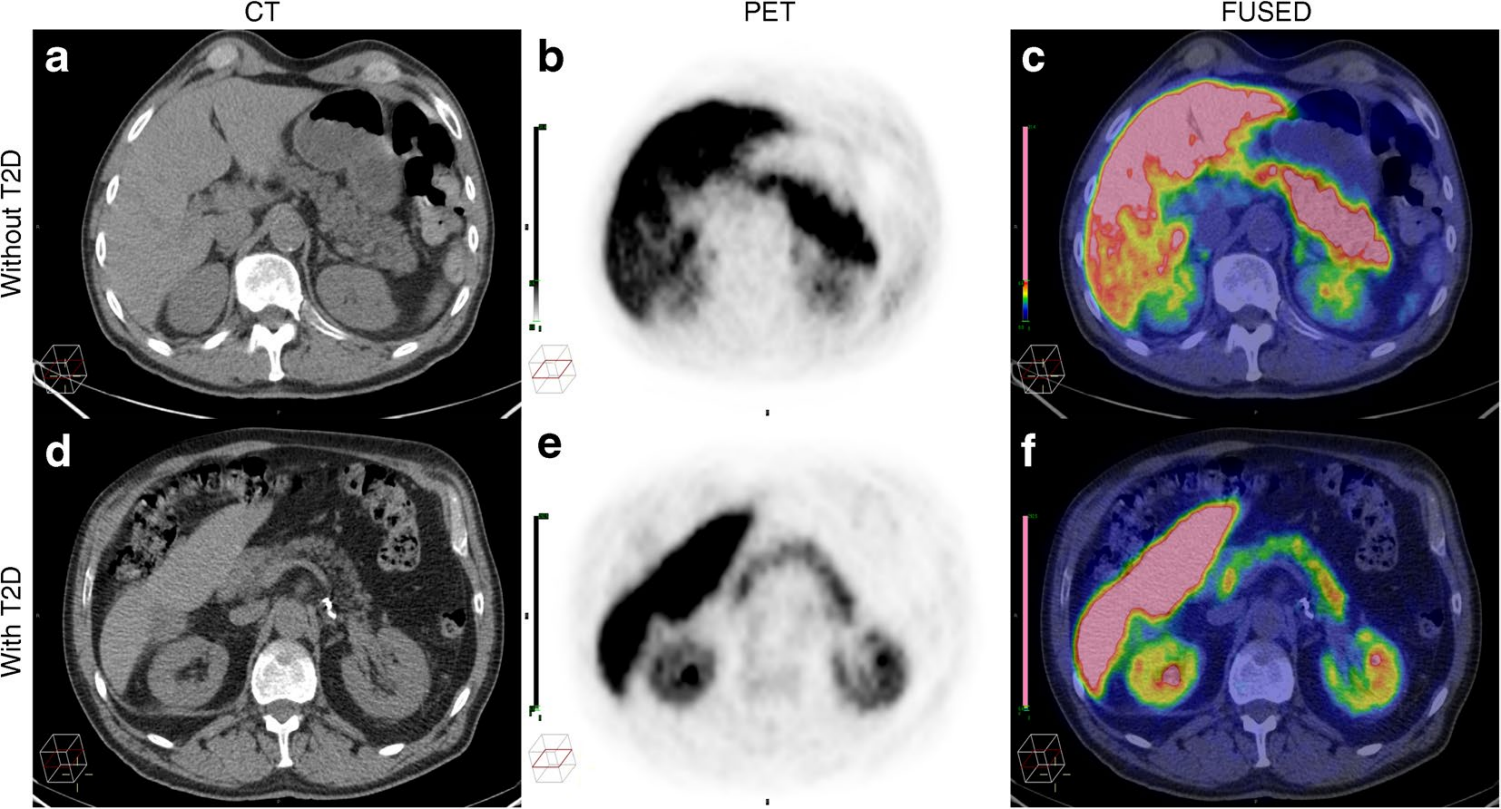
Images showing non-invasive imaging in two individuals, one without type 2 diabetes (**a**–**c**) and one with type 2 diabetes (**d**–**f**), using ^11^C-HED-PET/CT and CT. Distinct uptake of ^11^C-HED in the pancreas was seen in all examined individuals. Transverse CT (**a**, **d**), PET (**b**, **e**) and fused PET/CT (**c**, **f**) images in representative individuals. PET images are normalised to SUV = 6.6. T2D, type 2 diabetes

**Fig. 3:**
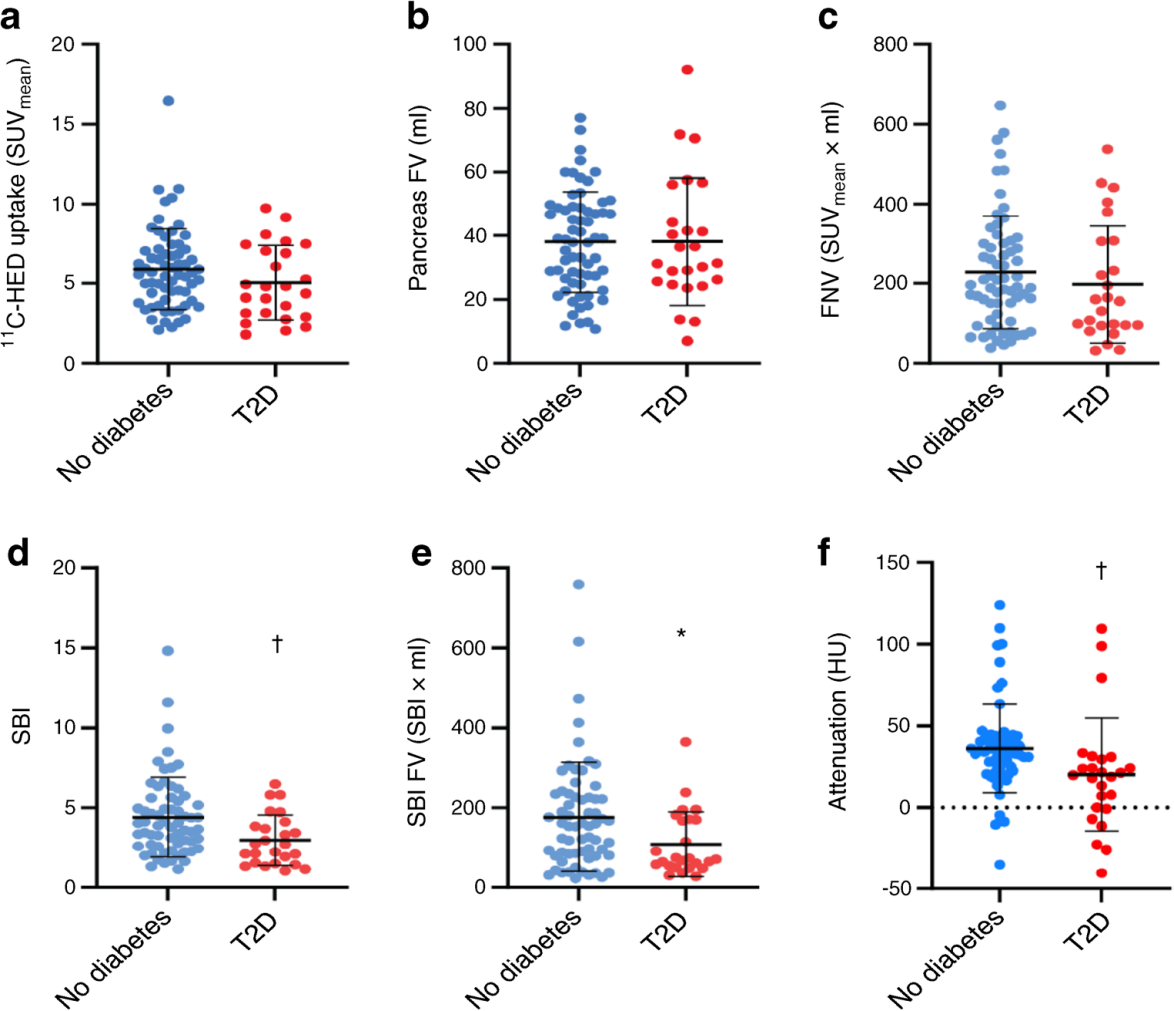
Plots showing quantification of uptake variables in the sympathetic innervation of the pancreas between individuals without diabetes (*n*=64) and individuals with type 2 diabetes (*n*=25). (**a**) ^11^C-HED uptake concentration per ml of the pancreas (SUV_mean_). (**b**) Volume of the pancreas (in ml). (**c**) The FNV of the pancreas (calculated as SUV_mean_ × FV [in ml]). (**d**) SBI of ^11^C-HED in the whole pancreas (ratio pancreas SUV_mean_ to left ventricle blood SUV_mean_). (**e**) SBI FV of the pancreas (calculated as SBI × FV [in ml]). (**f**) Attenuation of the pancreas (measured in HU). *p<0.05; ^†^*p*<0.01. T2D, type 2 diabetes

The uptake of ^11^C-HED in the pancreas was distinct in all examined individuals (Fig. 2a–f). The ^11^C-HED uptake was generally high in the abdominal organs with dense innervation. The ^11^C-HED uptake in the pancreas in individuals without diabetes was SUV_mean_ 5.9±2.5 (*n*=64, range 2.1–16.5), while the uptake in individuals with type 2 diabetes was SUV_mean_ 5.0±2.3 (*n*=25, range 1.8–9.7), with a weak tendency for lower ^11^C-HED uptake in the pancreas in individuals with type 2 diabetes (*p*=0.15; Fig. 3a). Uptake in the selected reference region, left ventricular blood, was SUV_mean_ 1.8±0.6 (*n*=64, range 0.8–3.4) and SUV_mean_ 1.4±0.5, (*n*=25, range 0.7–3.2), *p*=0.005, in individuals without diabetes and individuals with type 2 diabetes, respectively.

The pancreatic volume (FV) did not differ between the populations (individuals without diabetes 38.0±15.9 ml, individuals with type 2 diabetes 38.1±20.1 ml, *p*=0.73; Fig. 3b). Also, within the whole cohort and the two subgroups (i.e. with diabetes and without diabetes), no correlations were observed between FV and SUV_mean_, SBI, but the FNV (*r*=0.77, *p*<0.0001) and SBI FV (*r*=0.72, *p*<0.0001) correlated with FV (ESM Table 4). The FNV in the pancreas (i.e. the ^11^C-HED uptake multiplied by the pancreatic volume) and the total sympathetic innervation in the pancreas were similar between individuals without diabetes and individuals with type 2 diabetes (no diabetes 228.3±141.7 SUV × ml, type 2 diabetes 198.0±146.4 SUV × ml, *p*=0.30) (Fig. 3c). However, the SBI of ^11^C-HED in participants without diabetes (4.4±2.5, *n*=64, range 1.1–14.82) was higher than in those with type 2 diabetes (2.9±1.5, *n*=25, range 1.0–6.5, *p*=0.0049; Fig. 3d).

Adjusting the SBI values to the pancreatic volumes, that is, multiplying by the FV, rendered the SBI FV value, which is the specific binding of the tracer within the FV of the entire pancreas. The SBI FV within the whole pancreas in individuals with type 2 diabetes (108.6±80.6, *n*=25, range 27.0–365.7) was lower than in individuals without diabetes (177.5±136.5, *n*=64, range 22.3–758.8, *p*=0.0142; Fig. 3e). The attenuation of the pancreases in individuals with type 2 diabetes was decreased (no diabetes 36.3±27.0 HU, type 2 diabetes 20.3±34.7 HU, *p*<0.001; Fig. 3f), suggesting a higher degree of fat infiltration, as observed previously [28].

In all individuals (*n*=89), the FV of the pancreas correlated with the FNV (*r*=0.77, *p*<0.0001), the SBI FV (*r*=0.72, *p*<0.0001) and with CT attenuation (*r*=0.29, *p*=0.0060) (ESM Table 4). In all individuals (*n*=89), the CT attenuation of the pancreas correlated with SUV_mean_ (*r*=0.50, *p*<0.0001), SBI (*r*=0.60, *p*<0.0001), FNV (*r*=0.50, *p*<0.0001), SBI FV (*r*=0.57, *p*<0.0001) and with FV attenuation (*r*=0.29, *p*=0.0060) (ESM Table 5). Furthermore, in a subgroup of individuals, CT attenuation and FNV were utilised to see differences, which were analysed based on diet recommendations and pharmacological treatment (ESM Fig. 1).

### Pancreas size does not affect ^11^C-HED uptake

As mentioned, the pancreatic volumes in individuals with type 2 diabetes and individuals without diabetes were similar, although we observed a large variation in pancreatic size among the individuals with type 2 diabetes (Fig. 3b). Therefore, we subdivided the individuals with type 2 diabetes into those with a volume below or equal to the median (≤31.4 ml [SPV, *n*=13]) and those with a volume above the median (>31.4 ml [LPV, *n*=12]). However, both groups showed similar ^11^C-HED uptake (SUV_mean_) in the whole pancreas (SPV: SUV_mean_ 4.7±2.2; LPV: SUV_mean_ 5.4±2.3, *p*=0.43) and in the various pancreatic regions. The whole pancreatic and regional pancreatic ^11^C-HED uptake (SUV_mean_) were also similar between individuals without diabetes who had SPV and individuals without diabetes who had LPV (SPV: SUV_mean_ 4.95±1.74, *n*=25; LPV: SUV_mean_ 6.48±2.73, *n*=39, *p*=0.09). Thus, the ^11^C-HED uptake was independent of the size of the pancreas in both individuals without diabetes and those with type 2 diabetes.

### ^11^C-HED uptake in different segments of the pancreas

To evaluate the feasibility of assessing sympathetic innervation in different parts of the pancreas, we segmented the pancreas into the following regions: tail (cauda), body (corpus) and head (caput). These were further divided into two subregions, as described in Fig. 1b. As per the findings for the entire pancreas, there was a weak tendency for decreased ^11^C-HED uptake in individuals with type 2 diabetes in the different pancreas sections, especially the cranial head (*p*=0.18), distal body (*p*=0.18) and distal tail (*p*=0.19) (ESM Fig. 2a).

Regional differences in ^11^C-HED uptake between the pancreatic regions were observed in both individuals without diabetes (ESM Fig. 2b) and individuals with type 2 diabetes (ESM Fig. 2c). In the pancreas of individuals without diabetes, we observed higher ^11^C-HED uptake in the caudal head compared with the mean pancreas uptake (ESM Fig. 2b). Conversely, the ^11^C-HED uptake was lower in the proximal body and proximal tail. In individuals with type 2 diabetes, the ^11^C-HED uptake was lower in the cranial head and proximal body (ESM Fig. 2c). Further analyses of regional differences in ^11^C-HED uptake, pancreatic attenuation and FNV are listed in ESM Table 2. SBI measurements were also similar within subregions between individuals with diabetes and those without, except in the LPV subgroup, where individuals with diabetes had lower uptake. Additionally, within the subregions, the pancreatic caudal head had a higher SBI compared with the other subregions.

### Subgroup analyses suggest disease-related changes within pancreatic regions

We performed another subgroup analysis based on the type of treatment received by the individuals with type 2 diabetes, as this may be used to infer the level of endocrine function in the pancreas. The SUV_mean_ was lower in individuals with type 2 diabetes who received glucose-lowering drugs (oral glucose-lowering drugs and/or exogenous insulin) compared with those who were prescribed dietary and lifestyle changes (4.5±2.0 vs 6.1±2.5, *p*=0.15). Moreover, the pancreatic volumes (FNV) were smaller in individuals with type 2 diabetes receiving glucose-lowering drugs (33.1±18.8 vs 48.8±16.9 ml, *p*=0.01) (ESM Fig. 1).

## Discussion

In this retrospective analysis, we investigated the feasibility of repurposing the clinically available PET tracer ^11^C-HED to perform non-invasive assessments of the sympathetic innervation of the pancreas. ^11^C-HED demonstrated a distinct uptake in the pancreas of all examined individuals. Furthermore, the co-registered CT allowed for accurate delineation of the pancreatic volume as well as several anatomical subregions of the pancreas. Moreover, we were able to compare the uptake in individuals without diabetes to individuals with diabetes, as the individuals who underwent clinical assessment of PCC or PGL, or suspicion thereof, also included a subgroup of individuals with type 2 diabetes.

The worldwide prevalence of diabetes (both type 1 diabetes and type 2 diabetes combined), estimated in 2019 to be 9.3%, is increasing, leading to a global health burden on both individuals and healthcare systems [29]. Previous non-invasive imaging studies of the pancreas have focused on beta cells, which comprise 1–2% of tissue and are dispersed throughout the organ [30–37]. However, imaging beta cell mass in the pancreas in a diagnostic or prognostic fashion has proven difficult [38–40]. Therefore, in our present study, we focused on one common hypothesised co-factor in the onset of these diseases, the nervous system, to find a method for assessing intra-pancreatic sympathetic innervation for potential future use of the tracer ^11^C-HED in studies of diabetes aetiology, and possibly as a means for early diagnosis.

The nervous system has been implicated as an important factor in the development of both type 1 diabetes and type 2 diabetes [41, 42], and innervation and neuronal signals affect both pancreatic islet function and hormone release [43, 44]. In a recent study of pancreases from humans with type 2 diabetes, the pancreatic islet density of noradrenergic fibres was found to be increased in individuals with type 2 diabetes [45]. Thus, dysregulation of pancreatic islet innervation could contribute to both beta cell dysfunction and hormonal imbalance with hyperglucagonaemia in type 2 diabetes.

In the aetiology of type 1 diabetes, studies in mouse models have shown that neurons can affect immune cells and regulate beta cell inflammation through both sensory [2] and sympathetic nerve pathways [17]. Additionally, histological studies on pancreas sections from deceased human donors with type 1 diabetes have shown a lack of sympathetic nerves within islets [5], and individuals at risk of developing type 1 diabetes (i.e*.* presence of islet autoantibodies) have a lower density of these nerves in their islets [16].

Here, we demonstrated that ^11^C-HED-PET combined with CT can be used to assess sympathetic innervation in both the entire pancreas as well as in small, well-defined anatomical subregions. So far, innervation in the pancreas has not been systematically evaluated using PET technology in humans. Although other radiolabelled monoamine analogues have been used for pancreatic imaging, including [^11^C]5-hydroxytryptophan (^11^C-5-HTP) and [^18^F]fluoro-l-3,4-dihydroxyphenylalanine (DOPA), these imaging techniques have primarily focused on visualising the endocrine tissue in the pancreas, such as native islets of Langerhans, insulinomas or beta cell hyperplasia. We took advantage of the available state-of-the-art PET measurement techniques and quantified the FV by applying a VOI that automatically adjusts its size to include a predefined percentage of the maximum activity in the measured tissue, typically 41%, also referred to as A41 [27]. A41 is derived from tumour assessments on [^18^F]FDG-PET, whereby delineation by applying A41 was found to correspond best to the actual dimensions of the tumours on CT [27]. Even though this method cannot be generalised to other tracers, we explored choosing the 41% cut-off iso-contour to define the pancreatic FV with this tracer, ^11^C-HED. We found that the PET-VOI size of the pancreas correlated well with the size of the pancreas on CT, and was therefore considered feasible to apply in the present study. Further assessments of cut-off values will need to be performed in future prospective studies.

The uptake of ^11^C-HED varied between individuals, but the SUV_mean_ of the whole pancreas did not differ between the groups. However, the SBI and SBI FV were lower in those with type 2 diabetes than in individuals without diabetes. In this study, we opted for the left ventricular blood pool as a reference tissue for normalisation of the variability to determine the SBI. SBI is a valuable measurement, as it corrects the uptake in the tissue of interest (pancreas) for individual differences in tracer bio-distribution and metabolism, e.g. higher amounts of ^11^C-HED in the blood stream, which could conceivably lead to differences in tracer delivery or background binding.

The FNV was calculated as the product of the FV and the pancreatic SUV_mean_, as another measure of the sympathetic islet innervation. The FNV is assumed to represent the total amount of sympathetic innervation in the pancreas, rather than the concentration per volume unit. The pancreatic FV and FNV were found to be similar in individuals without diabetes and in those with type 2 diabetes. This is not surprising, as the criteria for the type 2 diabetes group allowed for broad inclusion, regardless of disease severity, age, time since diagnosis, weight, etc. This likely explains the large variability found in, for example, the pancreas volume in the type 2 diabetes group, as individuals may have been examined either early after diagnosis, or in a situation with longstanding disease (which may affect the pancreas volume differently). The other parameter that was clearly decreased in the type 2 diabetes group was pancreatic attenuation, likely corresponding to an increased infiltration of fat in the pancreatic tissue. Pancreatic fatty infiltration is related to diabetes, and previous studies have found that the pancreatic fat content is higher in individuals with type 2 diabetes [46]. In line with these findings, a growing body of knowledge indicates that non-alcoholic fatty pancreas disease may be associated with beta cell dysfunction, insulin resistance and inflammation, possibly leading to the development of diabetes and the metabolic syndrome [47]. The attenuation of fat on CT is approximately −100 HU, and that fatty infiltration, whether microscopic (fatty liver) or macroscopic (atrophic fatty pancreas), will consequently decrease the attenuation of the signal from the organ.

Our current study is limited in its observations by its retrospective design and missing data. Furthermore, we assume that the tracer in its entirety is internalised by the sympathetic nervous system, but there could be non-specific tracer uptake and, therefore, possible misinterpretations.

In this exploratory study, we wanted to investigate the sensitivity and granularity of the ^11^C-HED uptake in different parts of the pancreas. As such, we performed a division of the pancreas into six smaller segments, corresponding to the various pancreatic regions, as described in Fig. 2b. We chose to segment the pancreas into six subregions (instead of the traditional head, body and tail) for two reasons. First, we wanted to overcome a potential drawback in our study population where, in some individuals with diabetes, the tail region was difficult to delineate from the body, which resulted in only two regions. Additionally, we aimed to evaluate local innervation patterns in the pancreatic subregions, as it has been suggested that islets of Langerhans are spread out heterogeneously in the pancreas. Thus, beta cell dysfunction or dysregulation of innervation may conceivably affect different pancreatic subdivisions differently in the development of diabetes. Hence, further subdivision of the organ would provide more detailed information [48]. Here, we found no evidence of lower ^11^C-HED uptake in any specific subregion in individuals with type 2 diabetes compared with individuals without diabetes, even if there was a weak tendency for some segments. Assessment of SBI, on the other hand, did show a lower binding of the tracer in individuals with type 2 diabetes. Additionally, we found that there were small but reproducible differences within individual pancreases, where the head (caudal) exhibited higher innervation than the pancreas on average, while the body (proximal) had lower innervation.

### Conclusion

We demonstrate the feasibility of using ^11^C-HED PET for non-invasive assessment of pancreatic sympathetic innervation in humans, with potential differences in innervation in individuals with type 2 diabetes. These intriguing findings warrant further prospective evaluation, especially in patient cohorts with theoretical defects in the pancreatic sympathetic innervation, such as in type 1 diabetes.

### Supplementary Information

Below is the link to the electronic supplementary material.

Supplementary file1 (PDF 627 KB)

## Data Availability

The most relevant data generated or analysed during this study are included in this manuscript and the ESM. Further datasets used or analysed during the current study are available from the corresponding author upon reasonable request. Resources are also available upon request.

## Abbreviations

^11^C-HED: ^11^C-hydroxy ephedrine
CT: Computerised tomography
FDG: Fluorodeoxyglucose
FNV: Functional neuronal volume
FV: Functional volume
LPV: Large pancreatic volume
PCC: Pheochromocytoma
PET: Positron emission tomography
PGL: Paraganglioma
ROI: Region of interest
SBI: Specific binding index
SBI FV: Specific binding index with functional volume
SMV: Superior mesenteric vein
SPV: Small pancreatic volume
SUV: Standardised uptake value
VOI: Volume of interest
