# Supplementary material for: Non-invasive imaging of sympathetic innervation of the pancreas in individuals with type 2 diabetes
Source: Diabetologia. 2023 Nov 7;67(1):199–208. doi: 10.1007/s00125-023-06039-7 (PMC10709256; doi:10.1007/s00125-023-06039-7)
Supplement: Supplementary file 1 — Supplementary file1 (PDF 627 KB) [file 125_2023_6039_MOESM1_ESM.pdf]

Electronic Supplementary Material (ESM)

ESM Methods

^{11}C-Hydroxy ephedrine PET examination

A PET scanner (ECAT EXACT HR Plus, Siemens/CTI, Knoxville, Tenn., USA) was used from March 2000 to December 2004. Eleven PET examinations in eleven individuals were performed in two-dimensional mode with the axial field of view (FOV) of the PET scanner was 155 mm and the transaxial FOV was 585 mm. The individuals were placed supine on the scanner couch and a 2-min transmission scan was acquired for the localization of the diaphragm, which together with a previous contrast-enhanced CT was used to aid in positioning the tumor region within the FOV of the PET scanner. In this bed-position, a 10-min transmission scan was performed, for attenuation correction of the subsequent emission scan. Approximately 10–12 MBq/kg body weight (mean 574, range 91–943 MBq) of ^{11}C-HED was injected as an intravenous bolus and a dynamic 45-min examination sequence, comprising 14 time-frames, was started simultaneously. After reconstruction of PET data into transverse images. PET data, acquired 15-45 min after tracer injection, and were summed to create an average image volume.

ESM Results

^{11}C-HED uptake, CT attenuation (HU) and functional neuronal volume suggest disease related changes within pancreatic regions

Regional differences were analyzed utilizing parameters uptake, CT attenuation and functional neuronal volume (FNV). **ESM Table 1** shows comparisons between regions in without diabetes and individuals with type 2 diabetes.

Lower attenuation indicating fat infiltration with type 2 diabetes compared to without diabetes group

The pancreatic CT attenuation (whole pancreas) was lower in individuals with type 2 diabetes when compared to without diabetes individuals ($p<0.001$). Also, the CT attenuation was lower in individuals with type 2 diabetes than in without diabetes individuals when all six anatomical regions were individually tested pairwise ($p<0.001$), except for the distal tail ($p=0.131$) (**ESM Table 2**).

In the subgroup analyses of individuals with type 2 diabetes with SPV and LPV, pancreatic CT attenuation (HU) was similar between the groups (p=0.15).This was also true in the following regions; pancreatic head caudal (p<0.001), pancreatic head cranial (p<0.002), pancreatic body proximal (p<0.05), pancreatic body distal (p<0.001) and pancreatic tail distal (p<0.001).

ESM Tables

ESM Table 1.

Differences between with type 2 diabetes and without diabetes were analysed regarding ^{11}C-HED uptake (SUV), CT attenuation (HU), volume (ml) and functional neuronal volume (SUV x ml) *excluding distal tail; SUV = Standard uptake value; HU = Hounsfield Units; FNV = Functional neuronal volume.

Regions assessed	Individuals included	type 2 diabetes	Without diabetes	Parameters analyzed
**Whole pancreas**	All individuals with all observations	25	64	Volume , SUV, HU
**Regions 1-6**	All individuals with all observations	25	64	Volume , SUV, HU
**Regions 1-6**	All with complete observations	14	36	SUV, HU
**Regions 1-6**	All with complete observations	13	33	FNV
**Regions 1-5***	All with complete observations	24	60	SUV, HU
**Regions 1-5***	All with complete observations	21	55	FNV

ESM Table 2. Results of the analyses performed between with type 2 diabetes and without diabetes (Without diabetes).

	Included	Type 2 diabetes	Without diabetes		SUV	HU	FNV
**1**	Whole pancreas, All observations	25	64		0.153	**<0.001**	0.3002
**2**	Complete observations	14	36	In Without diabetes	**<0.001**	**<0.001**	
	Between regions 1-6			In type 2 diabetes	**<0.001**	**0.005**	
				Without diabetes+ type 2 diabetes combined	**<0.001**	**<0.001**	
				Without diabetes vs type 2 diabetes	0.801	0.177	
**3**	Complete observations	13	33	In Without diabetes			**<0.001**
	Between regions 1-6			In type 2 diabetes			**<0.001**
				Without diabetes+ type 2 diabetes combined			**<0.001**

				Without diabetes vs type 2 diabetes			0.947
**4**	Complete observations	24	60	In Controls	**<0.001**	**<0.001**	
	Between regions 1-5			In type 2 diabetes	**<0.001**	**0.003**	
				Without diabetes+ type 2 diabetes combined	**<0.001**	**<0.001**	
				Without diabetes vs type 2 diabetes	0.255	**0.002**	
**5**	Complete observations	21	55	In Controls			**<0.001**
	Between regions 1-5			In type 2 diabetes			**<0.001**
				Without diabetes+ type 2 diabetes combined			**<0.001**
				Without diabetes vs type 2 diabetes			0.801

ESM Table 3.

Sub-group analysis of pancreatic SUVmean and CT attenuation (HU) in individuals with type 2 diabetes with small pancreatic volume (SPV) ≤31.4 ml versus in those with large pancreatic

volume (LPV) >31.4 ml versus in individuals without diabetes (Without diabetes); * comparison between corresponding regions; ** comparison between corresponding regions and across regions; No. Individuals = number of individuals; WP = whole pancreas

**Sub-group Analysis**	**Type of observations**	**Regions assessed**	**Cohort 1 No. Individuals**	**Cohort 2 No. Individuals**
***SPV versus Without diabetes**	Complete observations	WP & 1-6	13	64
***LPV versus Without diabetes**	Complete observations	WP & 1-6	12	64
***SPV versus LPV**	Complete observations	WP & 1-6	13	12
****SPV**	Complete observations	1-6	13	13
****LPV**	Complete observations	1-6	12	12
****SPV , Excluding distal tail**	Complete observations	1-5	13	13
****LPV , Excluding distal tail**	Complete observations	1-5	12	12
***All observations SPV**	All observations	WP & 1-6	13	64
***All observations LPV**	All observations	WP & 1-6	12	64

ESM Table 4. Correlations of pancreas functional volume (FV) for all individuals, without and with type 2 diabetes.

**Parameter**	**r-value**	**p-value**
All individuals (n=89)		
SUVmean	0.19	0.075
Specific binding index	0.18	0.093
Functional neuronal volume	0.77	<0.0001 ‡
Specific binding index with functional volume	0.72	<0.0001 ‡
CT attenuation (HU)	0.29	0.0060 †
Without diabetes (n=64)		
SUVmean	0.18	0.16
Specific binding index	0.29	0.019 *
Functional neuronal volume	0.76	<0.0001 ‡
Specific binding index of the pancreas volume	0.77	<0.0001 ‡
CT attenuation (HU)	0.32	0.0098 †
With diabetes		
SUVmean	0.19	0.36
Specific binding index	0.21	0.32
Functional neuronal volume	0.78	<0.0001 ‡
Specific binding index of the pancreas volume	0.64	<0.001 §
CT attenuation (HU)	0.26	0.20

Correlations were computed for all study participants (n=89), and also separately for individuals with and without type 2 diabetes. All correlations were computed with a nonparametric Spearman test. * denotes a p-value of < 0.05, † denotes a p-value < 0.01 and ‡ <0.0001

ESM Table 5. Correlations of pancreas CT attenuation (HU) for all individuals, without and with type 2 diabetes.

**Parameter**	**r-value**	**p-value**
All individuals (n=89)		
SUV mean	0.50	<0.0001 ‡
Specific binding index	0.60	<0.0001 ‡
Functional neuronal volume	0.50	<0.0001 ‡
Specific binding index with functional volume	0.57	<0.0001 ‡
CT attenuation (HU)	0.29	0.0060 †
Without diabetes (n=64)		
SUVmean	0.50	<0.0001 ‡
Specific binding index	0.60	<0.0001 ‡
Functional neuronal volume	0.50	<0.0001 ‡
Specific binding index of the pancreas volume	0.57	<0.0001 ‡
CT attenuation (HU)	0.32	0.0098 †
With diabetes (n=25)		
SUV_{mean}	0.48	0.015*
Specific binding index	0.36	0.072

Functional neuronal volume	0.45	0.02*
Specific binding index of the pancreas volume	0.42	0.03*
CT attenuation (HU)	0.26	0.20

Correlations were computed for all study participants (n=89), and also separately for individuals with and without type 2 diabetes. All correlations were computed with a nonparametric Spearman test.* denotes a p-value of < 0.05, † denotes a p-value < 0.01 and ‡ <0.0001

ESM Figures

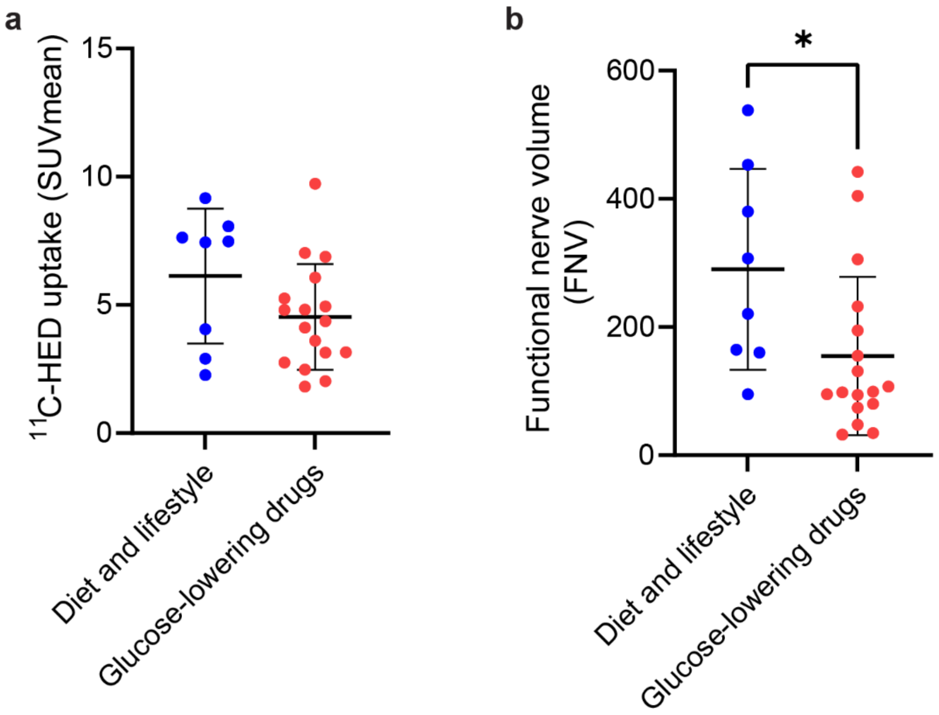

ESM Figure 1.

Plots showing uptake parameters in the sympathetic innervation of pancreas between individuals with and without diabetes to show sub-analysis of diet recommendations vs glucose-lowering drug treatment.

(a) Attenuation of the pancreas as measured by CT and (b) Functional Neuronal Volume of the pancreas (SUV_{mean} x FV)

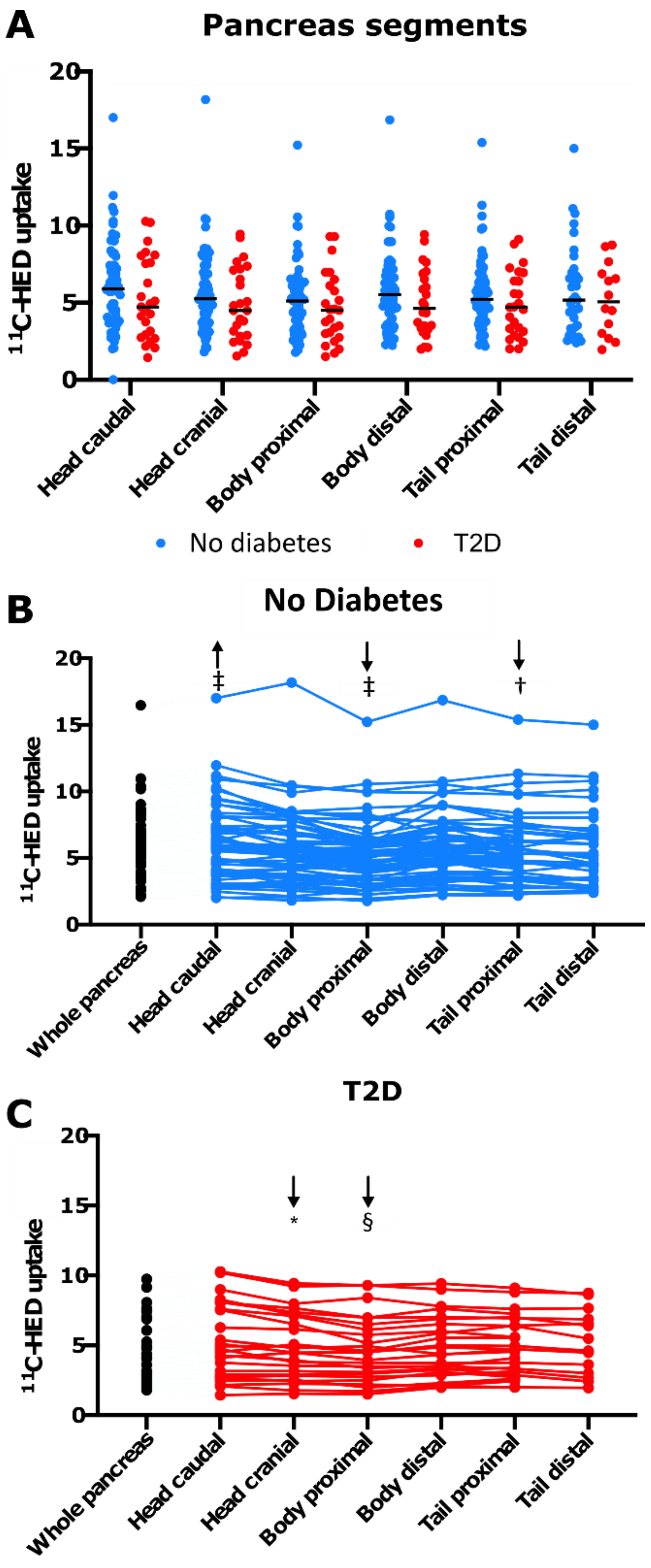
A
Pancreas segments
20
15
10
5
0
^{11}C-HED uptake
Head caudal
Head cranial
Body proximal
Body distal
Tail proximal
Tail distal
No diabetes
T2D
B
No Diabetes
‡
‡
†
Whole pancreas
C
T2D
*
§

ESM Figure 2.

^{11}C-HED uptake (SUV_{mean}) in the different pancreatic segments. (A) There were no differences in ^{11}C-HED uptake between without and with diabetes individuals in either of the pancreatic segments. (B) Plot showing the ^{11}C-HED uptake (SUV_{mean}) in each pancreatic segment for each individual without diabetes. (C) Plot showing the ^{11}C-HED uptake (SUV_{mean}) in each pancreatic segment for each individual with type 2 diabetes. * denotes a p-value of < 0.05, † denotes a p-value < 0.01, § denotes a p-value of <0.001 and ‡ denotes a p-value of <0.0001. Arrows indicate if uptake in a segment was decreased (arrow down) or increased (arrow up) compared to the whole pancreas.
